# Hand Preference Develops Across Childhood and Adolescence in Extremely Preterm Children: The EPICure Study

**DOI:** 10.1016/j.pediatrneurol.2019.04.007

**Published:** 2019-10

**Authors:** Neil Marlow, Yanyan Ni, Joanne Beckmann, Helen O'Reilly, Samantha Johnson, Dieter Wolke, Joan K. Morris

**Affiliations:** a Institute for Women's Health, University College London, London, UK; b Department of Psychology, University College Dublin, Dublin, Ireland; c Department of Health Sciences, University of Leicester, Leicester, UK; d Department of Psychology and Warwick Medical School, University of Warwick, Coventry, UK; e Population Health Research Institute, St George's, University of London, London, UK

**Keywords:** Extremely preterm, Laterality, Cognition, Handedness

## Abstract

**Aim:**

We attempted to determine how handedness changes with age and its relation to brain injury and cognition following birth before 26 weeks of gestation.

**Methods:**

We used data from the EPICure study of health and development following birth in the British Isles in 1995. Handedness was determined by direct observation during standardized testing at age 2.5, six, and 11 years and by self-report using the Edinburgh Handedness Inventory at 19 years. Control data from term births were included at six, 11, and 19 years.

**Results:**

In extremely preterm children left handedness increased from 9% to 27% between 2.5 and 19 years, with a progressive reduction in mixed handedness from 59% to 13%. Although individual handedness scores varied over childhood, the between-group effects were consistent through 19 years, with greatest differences in females. In extremely preterm participants, neonatal brain injury was associated with lower right handedness scores at each age and left-handed participants had lower cognitive scores at 19 years after controlling for confounders, but not at other ages.

**Conclusion:**

Increasing hand lateralization is seen over childhood in extremely preterm survivors, but consistently more individuals have non-right preferences at each age than control individuals.

**What this paper adds:**
1. After extremely preterm birth, handedness develops through to 19 years
2. Left or mixed handedness is more common at each age compared with controls
3. Neonatal brain injury is associated with increased left or mixed handedness
4. Cognitive impairment is only weakly associated with left handedness at 19 years

Hand preference may be easily and reliably assessed by direct examination using standardized presentation of everyday tasks. Around 85% to 90% of individuals in the general population demonstrate right-hand preference (RH). In contrast, studies reporting the laterality of populations of preterm children have frequently demonstrated an excess of non-RH, an observation that does not appear to be related to the presence or laterality of observed brain injury.^1^ In a recent systematic review, Domellöf and colleagues estimated the odds of preterm children being non–right handed compared with term-born children at 2.12 (95% confidence interval [CI]: 1.59 to 2.78).^2^ The authors identified that in some, but not all, studies there was concordance between non–right handedness and poorer neuropsychologic function. Thus it remains unclear whether this excess represented children with abnormal brain development and impaired performance or whether it was a specific feature of altered laterality in response to preterm birth. More recently in a neurocognitive evaluation of the Extremely Low Gestational Age Newborn cohort at age 10 years, left-handed and right-handed children performed similarly, but those with mixed handedness had greater odds of functional neurocognitive deficits.^3^ Although it is acknowledged that lateral preferences tend to be clear from age three years in the general population, there are no data on the evolution of laterality across childhood in preterm children to contextualize observations of lateral preferences at single ages or to determine whether associations with neuropsychologic functioning are constant at different ages. Such information may inform understanding of the different functional developmental organization of the brain after preterm birth, compared with typical development following normal gestation.

In the EPICure study, a longitudinal study of births in the United Kingdom and Ireland in 1995 at 25 completed weeks of gestation or less (extreme preterm [EP]), we have evaluated survivors through to 19 years of age. At each assessment, hand preferences were established as part of a multidomain assessment at home (2.5 years), school (six years and 11 years), or in a central London clinical research facility (19 years). In this article, we test three hypotheses: first, that hand preferences in the EP population are consistently more nonright compared with controls over the study assessments; second, that brain injury does not alter the distribution of hand preferences; and third, that the excess of nonright preferences among EP children is associated with lower intelligence quotient (IQ) and academic attainment.

## Methods

### Population

**Figure 1:**
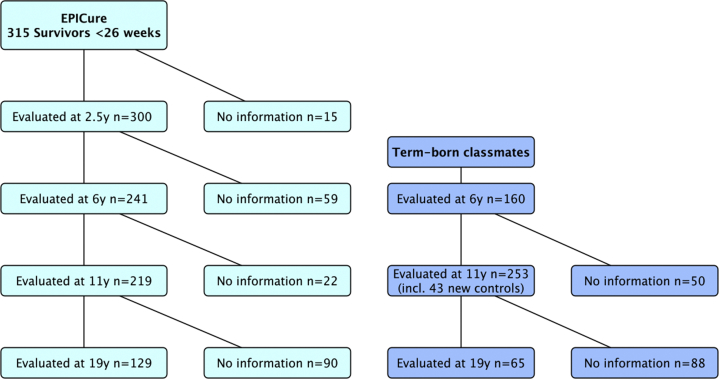
Disposition of the EPICure cohort and controls through age 19 years. The color version of this figure is available in the online edition.

The identification and perinatal outcomes for this cohort have been described previously,^4^ together with outcomes at 2.5,^5^ six,^6^ and 11 years.^7^ At 19 years we performed a center-based assessment at the Clinical Research Facility at University College Hospital, London.^8^ There was significant attrition between 2.5 (n = 300) and 19 years (n = 129; Fig 1). Term-born classmates of EP children were recruited at six (n = 160) and 11 years (n = 153), and those attending at 11 years were invited to the 19-year assessment (n = 65 attendees). Informed consent was obtained from parents up to 11 years and from individual participants at 19 years. Ethical approval was obtained *de novo* for each follow-up assessment. At 19 years approval was given by the South Central – Hampshire A Research Ethics Committee (Ref: 13/SC/0514).

### Methods

Hand preference was measured by direct observation of seven tasks as part of the home- or school-based assessment. Children were seated at a desk, and each task was presented in the midline with hands at rest away from the desk surface. The seven tasks comprised picking up a cube, placing a block on a tower, using a spoon, using a pen, using a crayon, pointing, and throwing a ball. If the child used more than one hand for each task, the full item set was repeated. Scores were +1 for use of right hand, −1 for left hand, and 0 where both hands were used. Scores with the left and right hand were totaled to give a range from 14 (complete RH) to −14 (complete left-hand preference [LH]). At 19 years, individual participants completed the Edinburgh Handedness inventory.^9^ From the original 10-item set, three items (using a knife, using a broom, and opening a box lid) were dropped as suggested by an analysis of internal consistency among items,^10^ leaving seven items that were summed to produce scores from 14 to −14 to match the earlier assessment scores.

Parental hand preference was determined by self-report at the time of the 2.5-year assessment on a five-point scale as “always” left or right, “mainly” left or right, and “use either hand” for all tasks.

Hand preference in our sample was defined *a priori* following visual inspection of the distribution of control child scores at 11 years (Supplementary Fig 1), the age at which controls were most frequently right handed and the latest age of direct observation. We defined groups as RH (+10 to +14) and LH (-14 to −10). Individuals scoring −9 to +9 were termed mixed handed (MH). These cutoffs were applied at each age to provide a consistent definition.

Data from the main study were combined to provide perinatal data, socioeconomic status (grouped as high, medium, and low), developmental outcome at 2.5 years (Mental Development Index, Bayley Scales of Infant and Toddler Development, Second Edition), IQ at six and 11 years (Mental Processing Composite, Kaufman Assessment Battery for Children), academic attainment at 11 years (Kaufman Assessment Battery for Children, Mental Processing Composite, standardized composite scores in reading and mathematics, Wechsler Individual Achievement Test Second Edition mathematics and reading composite) and 19 years (Full Scale IQ, Wechsler Abbreviated Scale of Intelligence, Second Edition), and the presence of cerebral palsy (with a Gross Motor Function Classification of ≥2 at any age). All outcome methodologies have been described previously.^4^, ^5^, ^6^, ^7^

### Statistical analysis

Mean handedness scores with 95% CI in EP participants and term-born controls were calculated at each time point stratified by the group variables.

Multilevel modeling was used to investigate trajectories of handedness scores from infancy to adulthood using Stata 14.2, treating the data as having a hierarchical structure with observations at each time point nested within each individual. This allows adjustment for missing observations where the individual was not assessed. In the analysis comparing EP and control groups, age was fitted as a random effect, which allows both the average level and the change in handedness score over age to vary between individuals. Age was centered at six years. A group term was added as a fixed covariate to test for a difference in intercept between the EP and control groups. An interaction term between age and group was then added to test whether the EP and control groups varied by slope, and then a quadratic function of age to test for curvature in the trajectories (Supplementary Table 1). The likelihood ratio test was used to evaluate the difference and compare the goodness of fit between models.

The effects of participant sex and socioeconomic status were examined by adding them separately to the model as fixed covariates and then as interactions with group (Supplementary Table 2). For a parameter to be retained in the model, it was required to have a *P* value < 0.05 in the likelihood ratio test. Analyses were first conducted in all participants with data available at any time point and then restricted to those with complete longitudinal data only.

Multinomial logistic regression models were used to estimate relative risk ratios (RRRs) of LH and MH preference to RH (the reference group) for EP participants at all ages. We then adjusted for participant sex in the models. Similar analyses were conducted within the EP group to test the effect of neonatal brain injury and parental handedness on handedness scores, with further adjustments for sex and gestational age. The risk for EP participants (controls as reference) was reported as RRRs with 95% CIs. Multiple linear regression models were used to analyze the effect of hand preference scores on cognitive score and reading and mathematics attainment in EP participants and controls, respectively. We adjusted for neonatal brain injury, gestational age and sex for EP participants, and sex for controls. Mean score differences between participants with LH/MH and RH and their 95% CIs were reported.

## Results

The EPICure cohort was evaluated at four ages. Progressive loss to follow-up occurred over the course of 19 years such that we retained 129 of the original 315 discharges or 42% of 306 long-term survivors at 19 years (Fig 1). Similar attrition occurred in the control population; 42% of those assessed at 11 years were also assessed at 19 years. A full dropout analysis has been published.^7^

In previous studies parental handedness has been shown to affect the development of hand preference. At 2.5 years, 89% of mothers of EP infants (n = 240) and 87% of fathers (n = 206) reported themselves as having RH, which is the expected population frequency. Only one mother reported that she was uncertain. We also asked parents to estimate their child's hand preference on the same scale. There was relatively poor agreement between this and our standardized observation (agreement: 44%; kappa: 0.227; *P* < .001; Supplementary Table 6).

### Changes in hand preferences over time

Hand preference was evaluated in all directly evaluated participants. Among the populations evaluated at each age, the distributions of hand preferences categorized into three groups (RH, MH, LH) changed between six and 19 years among EP participants and controls (Table 1). A similar pattern was observed whether all participants evaluated at each age, only those seen at each age point, or only participants without cerebral palsy were analyzed (Supplementary Fig 2). Compared with controls, among the EP group greater proportions with both MH preference and LH were seen at each age. At 2.5 years the majority of EP participants had MH (55%), only 32% had RH, and 10% had LH, as defined. Compared with controls, from six to 19 years EP participants were more likely to be left handed (at six years RRR, 7.2 [2.9 to 17.7]; at 11 years, 4.2 [2.0 to 8.8]; at 19 years, 4.2 [1.5 to 11.6]) and MH reduced in frequency (Table 1).

**Table 1:** Distribution of Hand Preferences Among Extremely Preterm Children and Controls at Each Assessment Point

Age	Extremely Preterm				Controls				Relative Risk Ratio (95% CI)∗	
	N	Right Handed (Score ≥ 10)	Mixed Handed (Score +9, −9)	Left Handed (Score ≤ −10)	n	Right Handed (Score ≥ 10)	Mixed Handed (Score +9, −9)	Left Handed (Score ≤ −10)	Mixed Handed	Left Handed
Cross-sectional analysis: all participants with at least one assessment										
2.5 years	277	89 (32.1%)	162 (58.5%)	26 (9.4%)		-	-	-	-	-
6 years	206	107 (51.9%)	62 (30.1%)	37 (18.0%)	159	125 (78.6%)	28 (17.6%)	6 (3.8%)	2.6 (1.5, 4.3)	7.2 (2.9, 17.7)
11 years	210	131 (62.4%)	36 (17.1%)	43 (20.5%)	152	129 (84.9%)	13 (8.5%)	10 (6.6%)	2.7 (1.4, 5.4)	4.2 (2.0, 8.8)
19 years	115	69 (60.0%)	15 (13.0%)	31 (27.0%)	62	47 (75.8%)	10 (16.1%)	5 (8.1%)	1.0 (0.4, 2.5)	4.2 (1.5, 11.6)
Longitudinal analysis: participants with all assessments excluding those with cerebral palsy										
2.5 years	91	29 (31.9%)	50 (55.0%)	12 (13.2%)		-	-	-	-	-
6 years	91	39 (44.3%)	34 (38.6%)	15 (17.1%)	54	42 (77.8%)	10 (18.5%)	2 (3.7%)	3.7 (1.6, 8.4)	8.1 (1.7, 37.6,)
11 years	91	54 (59.3%)	19 (20.9%)	18 (19.8%)	54	43 (79.6%)	8 (14.8%)	3 (5.6%)	1.9 (0.8, 4.7)	4.8 (1.3, 17.3)
19 years	91	51 (58.6%)	12 (13.8%)	24 (27.6%)	54	40 (78.4%)	6 (11.8%)	5 (9.8%)	1.6 (0.6, 4.6)	3.8 (1.3, 11.0)

Abbreviation:

CI = Confidence interval

∗ Multinomial logistic regression; reference category: right handedness.

**Figure 2:**
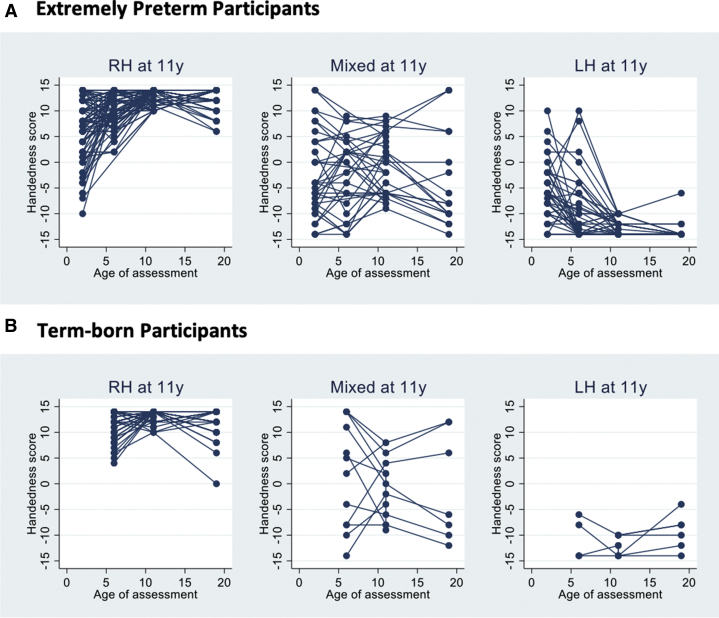
Change in individual hand preference scores in extremely preterm (Panel A) and control participants (Panel B) classified by predominant hand preference observed at 11 years for individuals seen at each age. Higher scores indicate greater right-hand preference. The color version of this figure is available in the online edition.

Centered on classification of preferences at 11 years, changes in hand preference scores over the study period are shown in Fig 2 and Supplementary Table 3. In the EP group at 2.5 years and in both groups at six years the spread of scores was broader than at 11 years indicating inconsistency in individuals. Self-assessment at 19 years produced a similarly broader range of scores.

**Figure 3:**
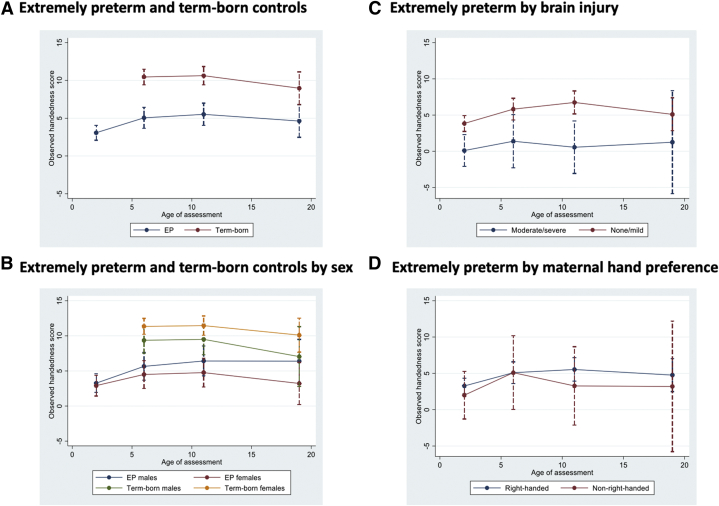
Multilevel modeling results (random slope model) for hand preference scores from 2.5 years through 19 years in extremely preterm children and controls. Panel A shows trajectories for extremely preterm and controls and Panel B for both groups by sex. Panel C show trajectories for extremely preterm participants with and without brain injury on neonatal ultrasound. Panel D shows trajectories for extremely preterm participants based on maternal self-report of hand preference. The color version of this figure is available in the online edition.

Multilevel modeling of handedness scores was used to account for loss to follow-up (Supplementary Table 2 and 4). Handedness scores tended to rise in all models to 11 years (Fig 3A). Differences in mean scores between the EP and control groups remained similar between six and 19 years in all participants. When grouped by sex, differences in scores were greatest among females (Fig 3B). Maternal (Fig 3D) and paternal hand preference (not shown) did not affect EP handedness scores.

### Effect of brain injury

Compared with EP participants with no brain injury on ultrasound or subependymal hemorrhage only, more severe neonatal brain injury was positively associated with a higher occurrence of LH and MH in EP participants at all ages except for 19 years (Supplementary Table 5; LH: RRR ranging from 2.9 to 4.2; MH: RRR ranging 2.3 to 2.6); these associations persisted after adjusting for sex and gestational age. In the model, handedness scores of EP participants with moderate or severe neonatal brain injury were on average 3.8 points below those of participants with no or mild brain injury (95% CI −6.1 to −1.4, *P* = 0.002) (Fig 3C).

### Association of hand preference with cognitive scores

IQ and academic attainment were significantly higher in controls compared with EP participants, as described previously.^6^ Among the EP group, scores tended to be higher in right compared with left or MH groups (Table 2). Significant differences from right-handed participants were only found among left-handed participants in IQ and reading at 11 years and in IQ at 19 years, but after adjustment for neonatal brain injury, sex, and gestational age only the IQ differences at 19 years persisted (adjusted difference in means: −6.8 points [95% CI: −13.2 to −0.3]).

**Table 2:** Hand Preferences and Cognitive/Attainment Scores for Extremely Preterm and Control Participants (Significant Differences in Means Shown in Bold)

Age	Test	Cognitive Scores			Unadjusted		Adjusted∗	
		Mean (S.D.)			Difference in Means from RH (95% CI)		Difference in Means from RH (95% CI)	
		Right Handed	Mixed Handed	Left Handed	Mixed Handed	Left Handed	Mixed Handed	Left Handed
Extremely preterm participants								
2.5 years	BSID2	82.3 (14.0)	81.4 (14.7)	80.9 (14.4)	−0.9 (−4.8, 3.0)	−1.5 (−8.3, 5.3)	0.6 (−3.3, 4.4)	−0.9 (−7.6, 5.7)
6 years	KABC	89.3 (13.0)	86.5 (14.1)	84.5 (13.8)	−2.9 (−7.1, 1.3)	−4.9 (−10.0, 0.2)	−1.8 (−5.9, 2.2)	−4.4 (−9.2, 0.5)
11 years	KABC	87.4 (14.4)	84.3 (16.4)	81.4 (18.4)	−3.0 (−9.0, 2.8)	**−6.0 (−11.4, −0.5)**	−1.7 (−7.4, 4.0)	−4.6 (−10.0, 0.7)
	Reading	84.3 (17.9)	80.2 (19.6)	76.6 (19.7)	−4.0 (−11.2, 3.1)	**−7.7 (−14.1, −1.2)**	−2.7 (−9.8, 4.4)	−6.1 (−12.7, 0.4)
	Mathematics	74.4 (19.1)	71.2 (21.6)	68.1 (21.9)	−3.2 (−10.8, 4.5)	−6.3 (−13.3, 0.7)	−1.7 (−9.3, 5.9)	−4.2 (−11.3, 2.9)
19 years	WASI-II	89.6 (14.6)	90.1 (16.1)	82.9 (14.0)	0.6 (−7.7, 8.9)	**−6.6 (−12.9, −0.3)**	0.5 (−7.9, 8.9)	**−6.8 (−13.2, −0.3)**
Control participants								
6 years	KABC	106.0 (11.9)	106.1 (11.8)	100.7 (9.0)	0.1 (−4.8, 4.9)	−5.3 (−15.1, 4.4)	0.0 (−4.9, 4.9)	−5.5 (−15.3, 4.4)
11 years	KABC	104.0 (11.0)	103.8 (13.9)	106.0 (9.7)	−0.2 (−6.6, 6.2)	2.0 (−5.2, 9.3)	−0.1 (−6.6, 6.3)	2.1 (−5.2, 9.4)
19 years	WASI-II	104.0 (10.0)	103.1 (6.6)	111.0 (14.6)	−0.9 (−7.8, 6.1)	7.0 (−2.4, 16.5)	−1.1 (−8.2, 6.0)	6.6 (−3.0, 16.2)

Abbreviations:

BSID2 = Bayley Scales of Infant and Toddler Development, Second Edition

CI = Confidence interval

EP = Extreme preterm

KABC = Kaufman Assessment Battery for Children

RH = Right-hand preference

WASI-II = Wechsler Abbreviated Scale of Intelligence, Second Edition

∗ EP group adjusted for neonatal brain injury, sex, and gestational age; control group adjusted for sex; multiple linear regression analysis.

## Discussion

Extremely preterm survivors more frequently have non-right preferences compared with controls. Using multilevel modeling to evaluate the changes over time, we demonstrated that the differences in handedness scores between EP and term-born controls persisted over 19 years. Among the EP group, handedness became progressively more polarized with age and MH became less frequent. Assessments showed variability in observed handedness particularly among EP individuals, with less variation in controls. EP participants who had evidence of intraventricular hemorrhage or periventricular leukomalacia were more likely to be non–right handed even after adjustment for important confounding variables, namely, sex and gestational age. The relationship between the excess of LH and MH in the EP group and lower neurocognitive scores appeared to be weak during childhood. It is notable that in our study after adjustment for confounders, IQ scores were significantly reduced by approximately 0.4 S.D. only at 19 years in participants with LH.

The trajectory of handedness has not been studied before in preterm populations, and indeed few studies have attempted to carry out direct observations of hand preference. It is commonly held that lateral preferences become apparent in the third year. At 2.5 years, when we might have expected preferences to be becoming clear, only 32% of EP children had RH and over half had mixed-handedness. Our data further suggest that, following extremely immature birth, these preferences are inconsistent over childhood, as shown in Fig 2, and the measures made at six and 11 years still demonstrate transition to more established preferences at 19 years—30% of those tested showed MH at six years, reducing to 21% at 11 years and 13% at 19 years. In contrast, LH increases in prevalence over the age range. The distribution of scores in the control group was closer to that expected in the general population. We used multilevel modeling to study the evolution of preferences over the four observations. Both extremely preterm and control groups showed similar trends to increasing right lateralization, but change was greater in the EP group (Supplementary Fig 1).

Most other studies have assessed handedness using parental questionnaires or accepting the preferred hand for writing. We were disappointed at the low agreement between handedness scores and parent report, which showed poor concurrent agreement at age 2.5 years (kappa 0.227). Of interest, using parent assessment at the first visit, the 11-year classification shows slightly better agreement (67.5%; kappa 0.412; Supplementary Table 6). It is a moot point as to which may be the more accurate reflection of “true” handedness. Our observations were conducted in a standardized fashion with identical presentation of tasks in the midline with both hands placed on the table. In contrast, parent report reflects accumulated observation, which may reflect bias in the presentation of tasks to one particular hand by the parent. Hence, we report direct standardized observation.

The relationship between handedness and measures of neurocognitive performance appear inconsistent across the literature.^2^ We hypothesized that this excess of non-RH among EP participants might be associated with poorer cognitive and education scores as has been shown in some other studies but were only able to demonstrate differences at 11 and 19 years and only among those who had LH rather than MH; moreover, only the 19-year findings correlated with measured IQ after adjustment for prespecified confounders and multiple comparisons. At 19 years attrition was highest, making this observation least reliable, although our modeling suggests that both hand preference distribution and cognitive scores remain stable. Interestingly, the magnitude of the difference in means was similar for controls at 19 years, although not of statistical significance, possibly because of the smaller sample size. This contrasts with the findings from the Extremely Low Gestational Age Newborn study where a range of lower scores appeared to be associated with MH preference, using parent-assigned hand preference.^3^

The strengths of our study lie in the longitudinal nature of the assessments and standardized testing undertaken during childhood. We used direct observation during a specific standardized test to classify hand preference in childhood and a well-validated self-completion questionnaire at 19 years. We reduced the number of variables used in our assessment in keeping with recommendations to provide a consistent scoring system in line with our direct observations. We suggest that direct measures may be more reliable than parent report, as used in other studies. In our study, we describe only poor to mild agreement between parent report and direct observation at 2.5 years. We used multilevel modeling to adjust for attrition during follow-up and adjusted for variables that *a priori* might confound any relationship. For example, key factors associated with cognitive scores in previous studies were male sex, lower gestation at birth, and evidence of brain injury. Likewise, males may show stronger right dominance than females in the general population. We further used continuous dimensional measures of handedness, to minimize the assumptions made during classification of hand preference. We chose a single measure of cognitive function at each age to avoid problems from multiple testing of related subscales and included standardized educational measures of mathematics and reading, which have been consistently shown to detect poorer performance in preterm populations.

The major weakness is the high level of attrition. However, the population that was not examined at 19 years showed few differences on a range of perinatal variables, and similar frequencies of hand preferences were seen in the smaller cohort evaluated at each time point, when compared with the full population examined at each age. Nonetheless, small differences might have biased these findings. We also chose to use the Edinburgh Handedness Inventory at 19 years because of time pressures within the face-to-face center-based evaluation schedule. Finally, there are no accepted formal definitions of laterality on testing. We chose cut points after inspection of control participant data at 11 years, the latest age at which we directly observed preferences, and applied it equally to define right and left handedness at each age for this study. Despite our *a priori* definition, it is possible that a different cut point may have altered the findings.

We have confirmed the excess of non-RH in a population of extremely preterm children through to young adult life. In our population we demonstrate the progression of lateralization over 19 years. Expected associations with cognitive test results were generally not found and restricted to those with LH; the significance and import of this observation remain obscure. Non-right laterality appears to be a relatively weak indicator of neurodevelopmental problems in the EP population.

Well-established handedness may reflect focused organization of the central nervous system. Magnetic resonance imaging studies suggest that the preterm brain is organized in a less focused fashion compared with the term brain, with wider activation during functional magnetic resonance imaging tasks^11^ and differences in connectivity.^12^ The poor lateralization of hand preference in this extremely preterm cohort may reflect this and provide explanation for this consistent finding.

## Supplementary data

Data Profile
