# Supplementary material for: Hand Preference Develops Across Childhood and Adolescence in Extremely Preterm Children: The EPICure Study
Source: Pediatr Neurol. 2019 Oct;99:40–6. doi: 10.1016/j.pediatrneurol.2019.04.007 (PMC6891894; doi:10.1016/j.pediatrneurol.2019.04.007)
Supplement: Supplementary Data [file mmc2.docx]

**Supplemental Materials**

**For :**

**Marlow N, Ni, Y, Beckmann J, O’Reilly H, Johnson, S, Wolke D, Morris J.**

**Hand preference develops across childhood and adolescence in extremely preterm children: The EPICure Study.**

**Table s1: Estimated mean differences in handedness scores and 95% CIs from multilevel modelling analyses in extremely preterm participants and term-born controls**

|  | ***Random intercept model* -**  Unadjusted model  (n=489) | ***Random slope model -***  Unadjusted model  (n=489) | ***Random slope model -***  Unadjusted model with EP*Age interaction  (n=489) | ***Quadratic model -***  Unadjusted model  (n=489) |
| --- | --- | --- | --- | --- |
|  | Estimate (95% CI) | Estimate (95% CI) | Estimate (95% CI) | Estimate (95% CI) |
| **Fixed** |  |  |  |  |
| Constant | 10.1 (8.9 to 11.3) * | 10.3 (9.1 to 11.5) * | 10.4 (9.2 to 11.5) * | 10.0 (8.9 to 11.2) * |
| EP | -5.9 (-7.5 to -4.4) * | -6.3 (-7.8 to -4.8) * | -6.3 (-7.8 to -4.8) * | -5.6 (-7.1 to 4.0) * |
| Age | 0.08 (0.03 to 0.13) * | 0.09 (0.03 to 0.14) * | 0.01 (-0.10 to 0.13) | 0.28 (0.18 to 0.37) * |
| EP*Age | - | - | 0.09 (-0.04 to 0.23) | - |
| Age*Age | - | - | - | -0.02 (-0.03 to -0.01) * |
|  |  |  |  |  |
| **Random** |  |  |  |  |
| Within-individual SD | 17.6 (15.8 to 19.5) | 13.9 (12.2 to 15.9) | 13.9 (12.2 to 15.9) | 13.3 (11.6 to 15.2) |
| Between-individual | - |  |  |  |
| Intercept SD | 62.9 (54.6 to 72.5) | 57.6 (49.9 to 66.5) | 57.5 (49.8 to 66.4) | 57.6 (49.9 to 66.4) |
| Slope SD | - | 0.09 (0.06 to 0.15) | 0.09 (0.05 to 0.15) | 0.10 (0.06 to 0.16) |
| corr(intercept, slope) | - | 1.4 (0.9 to 1.8) | 1.3 (0.9 to 1.8) | 1.3 (0.9 to 1.7) |

Note: Comparing the random intercept and random slope models using a likelihood-ratio test: $x^{2}$=78.75, P<0.001; the random slope model was selected over the random intercept model. Comparing the random slope and quadratic models using a likelihood-ratio test: $x^{2}$=24.49, P<0.001. To evaluate the fit of a quadratic model, we plotted the predicted trajectories from the two models alongside the observed values of handedness scores. Results from four individuals were given as examples (Figure S1). The figure showed that the improvement in fit appeared small for these individuals. Thus we reported the results using a random slope model.

**Table s2: Estimated mean differences in handedness scores and 95% CIs from multilevel modelling analyses in extremely preterm participants and term-born controls**

| Parameter | Unadjusted model  (n=489) | Adjusted for sex  (n=489) | Adjusted for SES  (n=465) |
| --- | --- | --- | --- |
|  | Estimate (95% CI) | Estimate (95% CI) | Estimate (95% CI) |
| **Fixed** |  |  |  |
| Constant | 10.2 (9.2 to 11.2)* | 10.6 (9.4 to 11.7)* | 10.1 (8.9 to 11.2)* |
| EP | -6.3 (-7.7 to -4.9)* | -6.2 (-7.7 to -4.8)* | -6.4 (-7.9 to -4.9)* |
| Age | 0.07 (0.01 to 0.12)* | 0.07 (0.01 to 0.12)* | 0.07 (0.01 to 0.12)* |
| Male | - | -0.9 (-2.3 to 0.5) | - |
| High SES | - | - | 0.2 (-1.3 to 1.7) |
|  |  |  |  |
| **Random** |  |  |  |
| Within-individual |  |  |  |
| EP SD | 16.3 (14.0 to 19.1) | 16.3 (14.0 to 19.1) | 16.5 (14.1 to 19.3) |
| Control SD | 6.0 (4.3 to 8.2) | 5.8 (4.2 to 8.0) | 5.9 (4.3 to 8.1) |
| Between-individual |  |  |  |
| EP Intercept SD | 71.6 (59.7 to 85.9) | 72.3 (60.2 to 86.7) | 72.2 (60.1 to 86.9) |
| EP Slope SD | 0.12 (0.07 to 0.20) | 0.12 (0.07 to 0.20) | 0.12 (0.07 to 0.21) |
| EP correlation (intercept, slope) | 2.1 (1.4 to 2.7) | 2.1 (1.4 to 2.8) | 2.1 (1.4 to 2.8) |
| Control Intercept SD | 45.0 (35.5 to 57.0) | 44.4 (35.0 to 56.3) | 47.0 (36.9 to 60.1) |
| Control Slope SD | 0.07 (0.03 to 0.16) | 0.07 (0.03 to 0.17) | 0.07 (0.03 to 0.17) |
| Control correlation (intercept, slope) | -0.04 (-0.63 to 0.56) | -0.02 (-0.62 to 0.58) | -0.04 (-0.66 to 0.59) |

*Note: the interaction terms of group with age, sex and SES were insignificant.*

**Table s3: Handedness scores in extremely preterm (EP) participants and term-born controls by age of assessment**

|  | **Age 2.5 years** | **Age 6 years** | | **Age 11 years** | | **Age 19 years** | |
| --- | --- | --- | --- | --- | --- | --- | --- |
|  | **EP (n=283)** | **EP (n=241)** | **Control (n=160)** | **EP (n=219)** | **Control (n=153)** | **EP (n=129)** | **Control (n=65)** |
| **Age of assessment** | | | | | | | |
| Mean (SD) | 2.5 (0.1) | 6.3 (0.5) | 6.1 (0.5) | 10.9 (0.4) | 10.9 (0.5) | 19.3 (0.5) | 19.2 (0.5) |
| Median (range) | 2.5 (2.3-3.3) | 6.3 (5.2-7.3) | 6.2 (5.1-7.2) | 10.9 (10.1-12.1) | 10.9 (9.8-12.3) | 19.3 (18.4-20.5) | 19.3 (18.1-20.3) |
| **Handedness Score** | | | | | | | |
| Mean (SD) | 3.1 (8.4) (n=277) | 5.0 (10.2) (n=206) | 10.5 (6.6) (n=159) | 5.5 (10.9) (n=210) | 10.6 (7.6) (n=152) | 4.9 (11.6) (n=115) | 9 (8.4) (n=62) |
| Difference in means (95%CI) | - | **-5.4 (-7.2 to -3.6)** | | **-5.1 (-7.1 to -3.1)** | | **-4.1 (-7.4 to -0.8)** | |
| **Sex** | | | | | | | |
| **Male** |  |  |  |  |  |  |  |
| % (n/N) | 47.7 (135/283) | 50.6 (122/241) | 44.4 (71/160) | 46.1 (101/219) | 41.8 (64/153) | 47.3 (61/129) | 38.5 (25/65) |
| Mean score (95%CI) | 3.3 (1.9-4.6) | 5.7 (3.7-7.6) | 9.4 (7.5-11.2) | 6.4 (4.3-8.5) | 9.5 (7.3-11.7) | 6.4 (3.3-9.5) | 7.0 (2.8-11.3) |
| **Female** |  |  |  |  |  |  |  |
| % (n/N) | 52.3 (135/283) | 49.4 (119/241) | 55.6 (89/160) | 53.9 (118/219) | 58.2 (89/153) | 52.7 (68/129) | 61.5 (40/65) |
| Mean score (95%CI) | 2.9 (1.4-4.4) | 4.5 (2.5-6.5) | 11.3 (10.2-12.5) | 4.8 (2.7-6.8) | 11.5 (10.1-12.8) | 3.2 (0.2-6.2) | 10.1 (7.7-12.5) |
| **Socioeconomic classification** | | | | | | | |
| **High** |  |  |  |  |  |  |  |
| % (n/N) | 31.0 (84/271) | 30.4 (66/217) | 35.2 (51/145) | 43.9 (79/180) | 55.8 (77/138) | 55.2 (69/125) | 60.9 (39/64) |
| Mean score (95%CI) | 3.8 (1.8-5.8) | 5.4 (2.9-8.0) | 10.5 (8.6-12.3) | 4.9 (2.4-7.9) | 10.8 (9.1-12.4) | 5.6 (2.8-8.4) | 9.5 (7.2-11.9) |
| **Medium** |  |  |  |  |  |  |  |
| % (n/N) | 34.3 (93/271) | 33.2 (72/217) | 39.3 (57/145) | 24.4 (44/180) | 16.7 (23/138) | 17.6 (22/125) | 23.4 (15/64) |
| Mean score (95%CI) | 3.9 (2.2-5.6) | 6.1 (3.6-8.6) | 10.1 (8.2-11.9) | 5.4 (2.1-8.7) | 12.3 (9.7-14.8) | 0.6 (-5.4 to 6.6) | 10.1 (4.9-15.4) |
| **Low** |  |  |  |  |  |  |  |
| % (n/N) | 34.7 (94/271) | 36.4 (79/217) | 25.5 (37/145) | 31.7 (57/180) | 27.5 (38/138) | 27.2 (34/125) | 15.6 (10/64) |
| Mean score (95%CI) | 1.6 (-0.1 to 3.2) | 4.5 (1.9-7.2) | 10.7 (8.5-12.9) | 5.8 (2.9-8.6) | 9.2 (6.2-12.1) | 5.4 (1.0-9.8) | 3.5 (-6.5 to 13.5) |
| **Gestational age** | | | | | | | |
| **24 weeks or less** |  |  |  |  |  |  |  |
| % (n/N) | 41.0 (116/283) | 40.3 (97/241) | **-** | 42.5 (93/219) | **-** | 40.3 (52/129) | **-** |
| Mean score (95%CI) | 2.5 (0.9-4.0) | 4.9 (2.7-7.1) | **-** | 5.1 (2.7-7.4) | **-** | 4.5 (0.9-8.0) | **-** |
| **25 weeks** |  |  |  |  |  |  |  |
| % (n/N) | 59.0 (167/283) | 60.0 (144/241) | **-** | 57.5 (126/219) | **-** | 56.7 (77/129) | **-** |
| Mean score (95%CI) | 3.5 (2.2-4.8) | 5.1 (3.3-7.0) | **-** | 5.8 (3.9-7.8) | **-** | 4.7 (2.0-7.5) | **-** |

| **Mother hand preference** | | | | | | | |
| --- | --- | --- | --- | --- | --- | --- | --- |
| **RH** |  |  |  |  |  |  |  |
| % (n/N) | 89.2 (240/269) | 88.2 (201/228) | **-** | 88.9 (185/208) | **-** | 90.3 (112/124) | **-** |
| Mean score (95%CI) | 3.3 (2.2-4.4) | 5.1 (3.6-6.6) | **-** | 5.5 (3.9-7.1) | **-** | 4.8 (2.5-7.0) | **-** |
| **Non-RH** |  |  |  |  |  |  |  |
| % (n/N) | 10.8 (29/269) | 11.8 (27/228) | **-** | 11.1 (23/208) | **-** | 9.7 (12/124) | **-** |
| Mean score (95%CI) | 2.0 (-1.3 to 5.3) | 5.1 (0.0-10.2) | **-** | 3.3 (-2.1 to 8.7) | **-** | 3.2 (-5.8 to 12.2) | **-** |
| **Father hand preference** | | | | | | | |
| **RH** |  |  |  |  |  |  |  |
| % (n/N) | 87.3 (206/236) | 88.4 (175/198) | **-** | 88.5 (162/183) | **-** | 92.5 (99/107) | **-** |
| Mean score (95%CI) | 3.5 (2.4-4.7) | 5.4 (3.8-7.0) | - | 5.4 (3.7-7.1) | - | 5.1 (2.7-7.5) | - |
| **Non-RH** |  |  |  |  |  |  |  |
| % (n/N) | 12.7 (30/236) | 11.6 (23/198) | - | 11.5 (21/183) | - | 7.5 (8/107) | - |
| Mean score (95%CI) | 2.1 (-1.1 to 5.3) | 6.1 (1.2-10.9) | - | 5.9 (0.4-11.4) | - | 6.9 (-4.5 to 18.3) | - |
| **Neonatal brain injury** | | | | | | | |
| **None/mild** |  |  |  |  |  |  |  |
| % (n/N) | 77.7 (220/283) | 22.4 (54/241) | - | 22.5 (49/218) | - | 17.2 (22/128) | - |
| Mean score (95%CI) | 3.8 (2.7-4.9) | 5.8 (4.3-7.3) |  | 6.7 (5.2-8.3) |  | 5.5 (3.1-7.8) |  |
| **Moderate/severe** |  |  |  |  |  |  |  |
| % (n/N) | 22.3 (63/283) | 77.6 (187/241) | - | 77.5 (169/218) | - | 82.8 (106/128) | - |
| Mean score (95%CI) | 0.1 (-2.1 to 2.3) | 1.4 (-2.3 to 5.1) |  | 0.6 (-3.1 to 4.2) |  | 1.3 (-5.9 to 8.4) |  |
| **Cerebral palsy** | | | | | | | |
| **Yes** |  |  |  |  |  |  |  |
| % (n/N) | 19.1 (54/283) | 20.3 (49/241) | **-** | 17.4 (38/219) | **-** | 7.8 (10/129) | **-** |
| Mean score (95%CI) | 2.9 (0.5-5.4) | 4.3 (-0.4 to 9.0) | **-** | 2.8 (-2.0 to 7.6) | **-** | 3.2 (-15.2 to 21.6) | **-** |
| **No** |  |  |  |  |  |  |  |
| % (n/N) | 80.9 (229/283) | 79.7 (192/241) | **-** | 82.6 (181/219) | **-** | 92.3 (119/129) | **-** |
| Mean score (95%CI) | 3.1 (2.0-4.2) | 5.1 (3.7-6.6) | **-** | 6.0 (4.4-7.5) | **-** | 4.7 (2.5-6.9) | **-** |
|  |  |  |  |  |  |  |  |
|  |  |  |  |  |  |  |  |

**Table s4: Estimated mean differences in handedness scores and 95% CIs from multilevel modelling analyses for extremely preterm participants only**

| Parameter | **Adjusted for neonatal brain injury (n=286)** | **Adjusted for gestational age (n=287)** | **Adjusted for mother hand preference**  **(n=267)** | **Adjusted for father hand preference (n=234)** | **Adjusted for plurality**  **(n=286)** | **Adjusted for oxygen status**  **(n=182)** | **Adjusted for cerebral palsy**  **(n=280)** |
| --- | --- | --- | --- | --- | --- | --- | --- |
|  | Estimate (95% CI) | Estimate (95% CI) | Estimate (95% CI) | Estimate (95% CI) | Estimate (95% CI) | Estimate (95% CI) | Estimate (95% CI) |
| **Fixed** |  |  |  |  |  |  |  |
| Constant | 4.9 (3.7 to 6.0) * | 4.5 (3.2 to 5.8) * | 3.0 (-0.1 to 6.1) | 3.0 (0.0 to 6.0) * | 4.3 (2.4 to 6.2) * | 5.0 (2.7 to 7.3) * | 4.0 (2.9 to 5.2) * |
| Age | 0.11 (0.04 to 0.18) * | 0.11 (0.03 to 0.18) * | 0.11 (0.03 to 0.18) * | 0.11 (0.03 to 0.19) * | 0.11 (0.03 to 0.18) * | 0.12 (0.04 to 0.20) * | 0.10 (0.03 to 0.18) * |
| Moderate-severe brain Injury | -3.8 (-6.1 to -1.4)* | - | - | - | - | - | - |
| Gestational age <25 weeks | - | -1.1 (-3.0 to 0.9) | - | - | - | - | - |
| Mother-RH | - | - | 1.2 (-2.1 to 4.4) | - | - | - | - |
| Father-RH | - | - | - | 1.4 (-1.8 to 4.6) | - | - | - |
| Plurality-single | - | - | - | - | -0.3 (-2.5 to 1.9) | - | - |
| In oxygen at 36 weeks | - | - | - | - | - | -0.8 (-3.5 to 2.0) | - |
| Cerebral palsy at 30 weeks | - | - | - | - | - | - | -0.8 (-3.4 to 1.8) |
|  |  |  |  |  |  |  |  |
| **Random** |  |  |  |  |  |  |  |
| Within-individual SD | 16.4 (14.0 to 19.1) | 16.4 (14.0 to 19.1) | 16.3 (14.0 to 19.1) | 16.2 (13.7 to 19.2) | 16.3 (14.0 to 19.1) | 15.3 (13.0 to 18.1) | 16.4 (14.1 to 18.1) |
| Between-individual |  |  |  |  |  |  |  |
| Intercept SD | 68.8 (57.3 to 82.6) | 71.2 (59.3 to 85.3) | 72.6 (60.2 to 87.5) | 71.2 (58.2 to 87.1) | 71.7 (59.8 to 86.1) | 75.0 (60.1 to 93.6) | 71.8 (59.7 to 86.2) |
| Slope SD | 0.12 (0.07 to 0.20) | 0.12 (0.07 to 0.20) | 0.12 (0.07 to 0.20) | 0.12 (0.07 to 0.22) | 0.12 (0.07 to 0.20) | 0.13 (0.08 to 0.23) | 0.12 (0.07 to 0.20) |
| Correlation (intercept, slope) | 2.0 (1.3 to 2.7) | 2.0 (1.4 to 2.7) | 2.1 (1.4 to 2.7) | 2.0 (1.2 to 2.7) | 2.1 (1.4 to 2.7) | 1.9 (1.1 to 2.7) | 2.1 (1.4 to 2.7) |

**Table s5: Hand preference and neonatal brain injury in extremely preterm participants**

| **Neonatal brain injury** | **Hand preference**  **n(%)** | | | **Unadjusted**  **RRR^+^ (95%CI)** | | **Adjusted for sex and gestation**  **RRR^+^ (95%CI)** | |
| --- | --- | --- | --- | --- | --- | --- | --- |
|  | **Right handed** | **Mixed handed** | **Left handed** | **Mixed handed** | **Left handed** | **Mixed handed** | **Left handed** |
| **2.5y** |  |  |  |  |  |  |  |
| Moderate/severe(n=57) | 10(17.5%) | 38(66.7%) | 9(15.8%) | **2.4(1.1, 5.1)** | **4.2(1.5, 11.9)** | **2.5(1.2, 5.4)** | **4.0(1.4, 11.5)** |
| None/mild(n=220) | 79(35.9%) | 124(56.4%) | 17(7.3%) |  |  |  |  |
| **6y** |  |  |  |  |  |  |  |
| Moderate/severe(n=36) | 12(33.3%) | 14(38.9%) | 10(27.8%) | 2.3(1.0, 5.4) | **2.9(1.1, 7.5)** | **2.4(1.0, 5.5)** | **2.9(1.1, 7.4)** |
| None/mild(n=170) | 95(55.9%) | 48(28.2%) | 27(15.9%) |  |  |  |  |
| **11y** |  |  |  |  |  |  |  |
| Moderate/severe(n=43) | 17(39.5%) | 10(23.2%) | 16(37.2%) | **2.6(1.1, 6.2)** | **3.9(1.8, 8.8)** | **2.5(1.0, 6.2)** | **3.9(1.7, 8.7)** |
| None/mild(n=166) | 113(68.1%) | 26(15.7%) | 27(16.3%) |  |  |  |  |
| **19y** |  |  |  |  |  |  |  |
| Moderate/severe(n=16) | 8(50.0%) | 2(12.5%) | 6(37.5%) | 1.2(0.2, 6.1) | 1.8(0.6, 5.7) | 1.1(0.2, 6.1) | 1.8(0.6, 5.8) |
| None/mild(n=91) | 60(61.2%) | 13(13.3%) | 25(25.5%) |  |  |  |  |

***^+^****: RRR (relative risk ratio) from multinomial logistic regression; reference category: no/mild neonatal injury in EP participants with right hand preference.*

**Table s6: Agreement between parent report of handedness at 2.5 years and direct observation at 2.5 and 11 years.**

|  | **Right Handed** | **Uncertain/mixed** | **Left handed** | **Percent agreement** | **Kappa (p)** |
| --- | --- | --- | --- | --- | --- |
| **2.5 year assessment (n=269)** |  |  |  |  |  |
| **Parent classification at 2.5y** | 58.9% | 8.5% | 32.6% | **43.8%** | **0.227 (p<.001)** |
| **Observation at 2.5y** | 32.1% | 58.5% | 9.4% |  |  |
|  |  |  |  |  |  |
| **11 year assessment (n=208)** |  |  |  |  |  |
| **Parent classification at 2.5y** | 58.2% | 8.7% | 33.2% | **67.5%** | **0.412 (p<.001)** |
| **Observation at 11y** | 62.4% | 17.1% | 20.5% |  |  |
|  |  |  |  |  |  |

**Figure s1: Distribution of lateral preference scores in extremely preterm participants and term-born controls at 11 years. *A priori* right and left handedness were defined as >10 and <-10, respectively.**

**Figure s2: Evolution of laterality from 2.5 to 19 years of age. Graph (a) demonstrates cross sectional data collected at each age and Graph (b) is restricted to only those seen at each assessment point, after exclusion of those with cerebral palsy**

**Figure s3: Predicted between-individual variance function for extremely preterm participants and term-born controls**

Variance function for EP group = 71.64 + 4.10*(Age-6) + 0.12*(Age-6)^2^

Variance function for control group = 44.99 – 0.07*(Age-6) + 0.07*(Age-6)^2^

The between-individual variance in handedness scores among EP participants is higher than among controls and it increases in both groups as they get older, but the increase is slower in the control group than in the EP participants.

|  |
| --- |
|  |
|  |
